# Exploration of bacteriophage therapy as a viable alternative to combat antibiotic-resistant bacterial infections: A comprehensive *in vitro* and *in vivo* evaluation

**DOI:** 10.6026/973206300212176

**Published:** 2025-07-31

**Authors:** Tarang Mehta, Saket Verma, Amberpreet Kaur Khangura, Niva Mahapatra, Khushboo Shrivastava, Sameer Gupta

**Affiliations:** 1 Department of Oral & Maxillofacial, Pathology and Oral Microbiology, K M Shah Dental College & Hospital , Sumandeep Vidyapeeth Deemed to be University, Vadodara, Gujarat, India; 2 Department of Biochemistry, Rajendra Institute of Medical Sciences Ranchi, Jharkhand 834009, India; 3 Department of Oral & Maxillofacial Pathology and Oral Microbiology, Dr Harvansh Singh Judge Institute of dental sciences and hospital, Panjab University, Chandigarh, India; 4 Department of Oral and Maxillofacial Pathology, Kalinga Institute of Dental Sciences, KIIT Deemed to be University, Bhubaneswar, Odisha, India; 5 Department of Pathology, Nalanda Medical College & Hospital, Patna-800026, Bihar, India; 6 Department of Oral & Maxillofacial Surgery, Inderprastha Dental College and Hospital, Sahibabad, Ghaziabad, Uttar Pradesh - 201010, India

**Keywords:** Bacteriophage therapy, multidrug-resistant bacteria, phage-antibiotic synergy

## Abstract

Antibiotic resistance in pathogens like *E. coli*, *Klebsiella pneumoniae*, and *Pseudomonas aeruginosa* poses a major threat to global health, with limited treatment options. This study systematically evaluated the efficacy of bacteriophage therapy *in vitro* and *in vivo* against multidrug-resistant strains. Phage treatment led to significant bacterial reduction (up to 4.5 log_10_ CFU/mL) and improved survival rates in mice (up to 85%). Synergistic effects with antibiotics and preservation of gut microbiota were observed without adverse reactions. These findings highlight phage therapy as a promising, targeted, and safe alternative for managing resistant infections.

## Background:

The emergence and proliferation of antimicrobial resistance (AMR) represents one of the most pressing challenges in contemporary medicine, threatening to return humanity to a pre-antibiotic era where common bacterial infections become life-threatening conditions [1]. The World Health Organization estimates that antibiotic-resistant bacteria are responsible for approximately 700,000 deaths annually worldwide, with projections reaching up to 10 million deaths per year by 2050 if no effective countermeasures are implemented [2]. This crisis has been exacerbated by the rapid evolution of bacterial resistance mechanisms that have outpaced the development of novel antimicrobial agents, creating an urgent need for alternative therapeutic strategies [3]. Among the most concerning resistant pathogens are multidrug-resistant (MDR) *Escherichia coli*, carbapenem-resistant *Klebsiella pneumoniae* (CRKP), and *Pseudomonas aeruginosa*, which have been designated as critical priority pathogens by the WHO due to their widespread resistance to last-resort antibiotics. These organisms are particularly problematic in healthcare settings, where they cause severe nosocomial infections including bacteremia, pneumonia, and urinary tract infections with mortality rates exceeding 40% in immunocompromised patients [4]. Bacteriophage therapy, first discovered in the early 20th century, has experienced a remarkable renaissance as researchers explore its potential to combat antibiotic-resistant infections. Bacteriophages (phages) are naturally occurring viruses that specifically target and lyse bacterial cells through sophisticated host recognition mechanisms, offering several distinct advantages over conventional antibiotics. Unlike broad-spectrum antibiotics, phages demonstrate exquisite specificity for their bacterial hosts, potentially minimizing disruption to beneficial microbiota and reducing the selective pressure for resistance development [5, 6, 7 8-9]. The self-replicating nature of phages provides a unique auto-dosing capability, where phage populations amplify at infection sites as long as susceptible bacteria are present. This property enables sustained therapeutic activity with potentially reduced dosing frequency compared to traditional antimicrobials. Furthermore, phages can target bacteria in biofilms, which are notoriously resistant to antibiotic penetration and represent a significant clinical challenge in chronic infections. Recent advances in phage engineering and genomic characterization have addressed many historical concerns about phage therapy, including standardization, safety, and regulatory approval pathways [5, 6-7]. The development of phage cocktails targeting multiple bacterial receptors has shown promise in reducing the emergence of phage-resistant variants, while synergistic combinations with antibiotics have demonstrated enhanced therapeutic efficacy [8, 9- 10]. Despite these promising developments, comprehensive clinical data evaluating the safety and efficacy of phage therapy against MDR pathogens remain limited. Most existing studies consist of case reports or small-scale trials, highlighting the need for systematic investigation of phage therapeutic potential across diverse bacterial targets and infection models [11, 12, 13, 14, 15-16]. Therefore, it is of interest to systematically evaluate and report the therapeutic potential of bacteriophage therapy against clinically significant multidrug-resistant bacterial pathogens using both *in vitro* and *in vivo* models.

## Materials and Methods:

## Bacterial strains and culture conditions:

Clinical isolates of MDR bacteria were obtained from the University Medical Center microbiology laboratory between January 2023 and March 2024. The study included 45 *E. coli* ST131 isolates, 38 CRKP isolates carrying various carbapenemase genes (KPC, OXA-48, NDM), and 42 *P. aeruginosa* isolates resistant to fluoroquinolones and β-lactams [6, 10 and 14]. Bacterial identification and antimicrobial susceptibility testing were performed using VITEK 2 systems (bioMérieux, France) following Clinical and Laboratory Standards Institute guidelines. All isolates demonstrated resistance to at least three different antibiotic classes. Bacteria were cultured in Luria-Bertani (LB) broth at 37°C with constant agitation at 180 rpm. Optical density measurements at 600 nm (OD_600_) were used to standardize bacterial concentrations, with 0.5 McFarland units corresponding to approximately 1.5 x 10^8^ CFU/mL [11].

## Bacteriophage isolation and purification:

Phage isolation was conducted from hospital wastewater, sewage samples, and environmental water sources collected from five different geographic locations [6, 17]. Samples were filtered through 0.45-µm membranes and processed using standard enrichment protocols. Target bacteria were grown to mid-log phase (OD_600_ = 0.4-0.6) before phage screening using the standard spot test assay [11]. Purification involved multiple rounds of plaque purification followed by cesium chloride density gradient centrifugation. Phage titers were determined using the double-layer agar method and expressed as plaque-forming units per milliliter (PFU/mL) [9]. High-titer phage stocks (>10^9^ PFU/mL) were stored at 4°C in SM buffer supplemented with chloroform.

## Phage characterization:

Morphological analysis was performed using transmission electron microscopy (Zeiss EM 10C) with negative staining. Host range determination involved testing purified phages against all bacterial isolates using standardized spot tests with phage concentrations of 106 PFU/mL [11, 17]. One-step growth curves were conducted to determine latent periods and burst sizes. Exponentially growing bacteria (108 CFU/mL) were infected with phages at multiplicity of infection (MOI) of 0.1, and samples were collected at 5-minute intervals for 120 minutes [17]. Stability testing evaluated phage viability across pH ranges (2-12) and temperatures (4-80°C) over various time periods. Genomic sequencing was performed using Oxford Nanopore and Illumina platforms, with bioinformatic analysis conducted to identify potential virulence factors, antibiotic resistance genes, and lysogeny markers [6, 14].

## *in vitro* efficacy studies:

Bactericidal activity was assessed using time-kill assays with bacterial suspensions (10^6^ CFU/mL) exposed to phages at MOI ratios of 0.001, 0.01, 0.1, and 1.0 [17]. Samples were collected at predetermined intervals and plated for viable cell counts. Phage susceptibility testing followed standardized protocols with 20 µL phage applications on bacterial lawns [11]. Biofilm studies utilized 96-well polystyrene plates with established 24-hour biofilms exposed to phage treatments. Crystal violet staining quantified biofilm biomass, while confocal laser scanning microscopy assessed biofilm architecture and bacterial viability [2].

## *in vivo* animal studies:

All animal experiments were approved by the Institutional Animal Care and Use Committee (IACUC Protocol #2023-089). Male C57BL/6 mice (8-10 weeks, 20-25g) were obtained from Charles River Laboratories and acclimatized for one week before experimentation.

## Infection models:

(1) systemic bacteremia via intra-peritoneal injection of 10^7^ CFU; (2) urinary tract infection through transurethral catheterization; and (3) pneumonia via intranasal instillation [7, 10]. Neutropenia was induced using cyclophosphamide (150 mg/kg) administered 3 days before infection to simulate immunocompromised conditions [10]. Phage therapy was administered at 5 x 10^9^ PFU via intraperitoneal injection at 0, 12, and 24 hours post-infection. Control groups received sterile phosphate-buffered saline. Survival was monitored for 14 days, with bacterial loads quantified in blood, urine, lungs, kidneys and spleen at predetermined endpoints [10].

## Statistical analysis:

Data analysis was performed using GraphPad Prism 9.0 software. Survival curves were analyzed using Kaplan-Meier methods with log-rank tests. Bacterial load comparisons utilized unpaired t-tests or one-way ANOVA with Tukey's multiple comparison tests. Statistical significance was defined as p < 0.05, with all experiments performed in triplicate.

## Results:

We successfully isolated 23 distinct bacteriophages from environmental samples, with 12 demonstrating significant lytic activity against target MDR bacteria. Based on morphological and genomic analyses, the selected phages belonged to the *Tequatrovirus* genus (*Straboviridae* family) for *E. coli*-targeting phages, *Drulisvirus* genus for *K. pneumoniae*-targeting phages, and *Pbunavirus* genus for *P. aeruginosa*-targeting phages [6, 14 and 17]. Host range analysis revealed that phage cocktails achieved coverage of 94.4% of *E. coli* ST131 isolates, 78.9% of CRKP isolates, and 83.3% of *P. aeruginosa* isolates [17]. One-step growth curves showed optimal latent periods of 15-25 minutes and burst sizes ranging from 161-473 PFU per infected cell [6]. Stability test demonstrated phage viability across pH 4-8 and temperatures up to 40°C for extended periods. Genomic analysis confirmed the absence of lysogeny-associated genes, antibiotic resistance determinants, or known virulence factors in all therapeutic phage candidates [6, 14]. Time-kill assays demonstrated rapid bacterial reduction within 2-4 hours of phage exposure across all tested MOI ratios. Table 1 (see PDF) summarizes the bacterial load reductions achieved at 8 hours post-treatment. Biofilm disruption studies showed significant biomass reduction with phage treatment compared to controls. *E. coli* biofilms demonstrated 78% reduction, *K. pneumoniae* showed 65% reduction, and *P. aeruginosa* achieved 58% biomass reduction following 24-hour phage exposure [2]. Animal survival studies revealed dramatic improvements in phage-treated groups across all infection models. Table 2 (see PDF) presents survival data and bacterial burden analysis. Combination therapy studies using phages with sub-inhibitory antibiotic concentrations demonstrated synergistic effects. The combination of phage cocktails with ceftazidime-avibactam against CRKP produced a 10^5^-fold reduction in bacterial burden in both cecum and kidney tissues (p < 0.001) [10]. Comprehensive safety evaluation revealed no significant adverse effects from phage therapy. Histopathological examination of major organs showed no treatment-related pathological changes. Proinflammatory cytokine analysis (IL-1β, TNF-α, IL-6) demonstrated no significant elevation compared to control groups [7]. Microbiome analysis using 16S rRNA sequencing showed preservation of beneficial bacterial communities in phage-treated animals, contrasting with significant dysbiosis observed in antibiotic-treated controls [9, 15]. Bacterial resistance to phages emerged in approximately 0.1-0.3% of treated populations after 72 hours of exposure. Notably, phage-resistant mutants demonstrated increased susceptibility to serum killing and reduced virulence in secondary infection models [10]. Sequential phage application and cocktail rotation strategies effectively minimized resistance development [18].

## Discussion:

This comprehensive study provides robust evidence supporting the therapeutic potential of bacteriophage therapy against clinically significant MDR bacterial pathogens. Our findings demonstrate that carefully selected and characterized phage cocktails can achieve substantial bacterial load reductions both *in vitro* and *in vivo*, with safety profiles that compare favorably to conventional antimicrobial agents. The bacterial load reductions observed in our study (3.5-4.2 log_10_ CFU/mL) are consistent with previous reports and exceed the clinical threshold for therapeutic significance [1, 2]. The rapid onset of bactericidal activity within 2-4 hours represents a significant advantage over antibiotics, which may require 24-48 hours to achieve comparable effects. This rapid action could be particularly beneficial in sepsis management, where time-to-effective-therapy directly correlates with patient outcomes. Our *in vivo* survival data (75-85% in treated groups versus 15-25% in controls) demonstrate the life-saving potential of phage therapy in severe infection models. These results are particularly compelling given that we utilized immunocompromised animal models that more accurately reflect the clinical populations most vulnerable to MDR infections [10]. The observed therapeutic efficacy across multiple infection sites (systemic, pulmonary and urogenital) suggests broad clinical applicability. The synergistic effects observed with phage-antibiotic combinations align with emerging evidence supporting combination therapies [10, 16]. The 10^5^-fold bacterial reduction achieved with phage-ceftazidime-avibactam combinations suggests that phages may restore antibiotic susceptibility in resistant organisms, potentially extending the utility of existing antimicrobial agents. This finding has profound implications for clinical practice, as it offers a pathway to overcome resistance without requiring novel antibiotic development. Safety assessment represents a critical consideration for clinical translation of phage therapy. Our comprehensive evaluation, including histopathology, cytokine analysis, and microbiome assessment, revealed no significant adverse effects [7]. The preservation of beneficial microbiota represents a substantial advantage over broad-spectrum antibiotics, which frequently cause dysbiosis and secondary complications such as *Clostridioides difficile* infections [9, 15]. The emergence of phage resistance in 0.1-0.3% of bacterial populations is concerning but manageable through established strategies [18]. The observation that phage-resistant mutants demonstrated reduced virulence and increased serum susceptibility suggests that resistance may impose fitness costs that potentially reduce pathogenicity [10]. Cocktail rotation and sequential therapy protocols can further minimize resistance development. Several limitations warrant consideration. Our study utilized specific bacterial isolates from a single geographic region, which may limit generalizability to different resistance patterns or bacterial populations. The animal models, while clinically relevant, cannot fully recapitulate the complexity of human infections, particularly in immunocompromised patients with multiple comorbidities. Additionally, the optimal dosing regimens and treatment durations for human applications require further investigation. The regulatory landscape for phage therapy continues to evolve, with recent FDA guidance providing clearer pathways for clinical development [8]. Our standardized characterization protocols and safety data contribute to the evidence base needed for regulatory approval. The personalized medicine approach inherent to phage therapy, where specific phages are selected based on bacterial susceptibility profiles, aligns with contemporary precision medicine paradigms [16]. Future research directions should focus on large-scale clinical trials, optimization of delivery methods and development of rapid diagnostic platforms to enable real-time phage selection [3]. The integration of artificial intelligence and machine learning approaches may enhance phage-host matching and predict optimal treatment regimens [19, 20].

## Conclusion:

Bacteriophage therapy demonstrated potent antibacterial activity against multidrug-resistant pathogens with high safety and efficacy in both *in vitro* and *in vivo* models. Its synergism with antibiotics and minimal impact on host microbiota make it a viable alternative to conventional treatments. With further clinical validation, phage therapy holds strong potential as a precision tool to combat antibiotic resistance.
